# Stages of organizational development and employee assistance programs in Taiwan

**DOI:** 10.1057/s41599-023-01567-4

**Published:** 2023-03-07

**Authors:** Yin-Che Chen, Yun-Chu Chen, Hui-Chuang Chu

**Affiliations:** Department of Educational Psychology and Counseling, National Tsing Hua University, Hsinchu, Taiwan

**Keywords:** Business and management, Health humanities

## Abstract

Employee assistance programs (EAPs) provide work, living, and health services to help employees overcome personal and organizational obstacles that affect their productivity. Most businesses in Taiwan are small or medium-sized, and their scale, stage of development, and resources affect their implementation of EAPs. This study explored EAPs and related measures that organizations can implement in each stage of their development. The results may serve as a reference for human resources personnel in planning EAPs, specifically in identifying appropriate measures to implement for each developmental stage of their organizations. The modified Delphi method and fuzzy analytic hierarchy process were used to organize and analyze key EAP measures and their weights during the creation, guidance, authorization, coordination, and collaboration stages of organizational development. Data analysis revealed that in all five stages of organizational development, work-related EAP measures are the most crucial. As an organization transitions from the creation to collaboration stages, the work dimension is neglected in favor of the health dimension. In the authorization stage, the organization begins to provide a wider range of services in the living dimension. The results and other information regarding EAP service models indicate that in each developmental stage, an organization should adopt a different EAP service model that suits its resources, organizational structure, implementation of EAP measures, and other factors.

## Introduction

Employee assistance programs (EAPs) provide work, living, and health services to help employees overcome personal and organizational obstacles that affect their productivity and performance at work (Ministry of Labor, Executive Yuan, 2017). EAPs are usually better received and more widely accepted by employees than other management strategies. EAPs play a key role in maintaining organizational stability. Employees are crucial to the sustainable development and operations of enterprises, and EAPs support their professional development. EAPs also help employees develop professionally as their enterprises evolve. Besides, new researches provide evidence that the use of brief counseling from EAPs is associated with improvements in clinical and work performance domains and organizational benefits widely (Attridge, 2019; Compton and McManus, 2015). In addition, according to the Workplace Outcome Suite Annual Report in 2021, employees who used EAP counseling increased significantly in work-related outcomes. Similar findings were consistently obtained across many contexts even given the challenges of delivering counseling during the COVID-19 global pandemic (LifeWorks, 2022).

EAP-related activities in Taiwan began with the Young Christian Workers, a group established by a Christian church in 1958 to help Church members adapt to workplace stress. In the 1970s, companies began to implement measures related to work–life balance counseling. In 1972, Panasonic Taiwan assigned experienced and enthusiastic female personnel to serve as mediators of communication between supervisors and operators. In 1976, TECO Electric & Machinery Company established the “Love Bridge,” a mailbox for communication and expressing feelings. In 1979, the China Youth Corps established “Teacher Chang” and the Industry and Commerce Youth Service Teams, which provide psychological counseling services and mental health education. In 1980, CPC Corporation hired professionals to provide counseling services to its employees. After the construction of Hsinchu Science Park in 1988, the electronic component and personal computer manufacturers comprising Taiwan’s information technology (IT) industry became internationally competitive. Companies such as Taiwan Semiconductor Manufacturing Company, United Microelectronics Corporation, Macronix International, and Acer also contributed to advancing IT. Government agencies have striven to implement labor counseling services. In 1992, the Council of Labor Affairs of the Executive Yuan launched a labor counseling service called the “Industrial Social Work” program. In 1994, an EAP was officially launched to provide employee assistance. In 2000, the Hsinchu Lifeline Association established the first external EAP center, and EAPs began to diversify. In 2009, the Executive Yuan extended EAPs to the public sector. EAPs have become crucial strategic tools for enterprise management and crucial welfare measures for public and private institutions in Taiwan. To encourage enterprises to implement EAPs, the Ministry of Labor provides them with access to training courses, enterprise experts, and guidance (Ministry of Labor, Executive Yuan, 2021).

Most studies on EAPs have focused on the positive effects of EAPs on organizational commitment (Lee et al., 2008; Lin, 2014) and organizational performance (Lin et al., 2017; Sun, 2001). A few studies have examined EAPs in relation to organizational development and reported that enterprises often have different perceptions of and adopt different measures related to the content and implementation of EAPs depending on their size and amount of capital (Chen, 2003; Wilson et al., 1999). However, none have examined EAPs in relation to the organizational development process (Chen, 2004; Li et al., 2016; Lin et al., 2017). Because of this lack of research, a clear set of guidelines regarding what EAP measures enterprises should implement at different stages of organizational development has not yet been established.

Although numerous studies have provided evidence of the positive effects of EAPs on enterprise performance, the number of Taiwanese enterprises willing to invest in EAPs remains low (Chen, 2005). This can be explained by the composition of Taiwanese enterprises. In 2018, small and medium-sized enterprises accounted for 97.7% of all enterprises in Taiwan (Ministry of Economic Affairs, Executive Yuan, 2018), and research has demonstrated that small enterprises typically consider providing workplace health services difficult because they lack professional knowledge and skills (Chen, 2005). Thus, factors associated with the limited scale of small enterprises are the main obstacles preventing Taiwanese enterprises from investing in EAPs (Chen, 2003). The main users and direct beneficiaries of EAPs are employees; however, because each employee has different needs and because enterprises are limited by practical constraints, enterprises cannot identify and satisfy the needs of every employee. This study identified the most appropriate EAP services for enterprises to implement at each stage of organizational development. EAP reference indicators were developed using the modified Delphi method and fuzzy analytic hierarchy process. The results serve as a reference for enterprises formulating EAP measures.

## Literature review

### EAPs

EAPs were originally developed to address alcohol abuse problems among employees (Ministry of Labor, Executive Yuan, 2017). Since then, EAPs have grown to encompass a wider range of employee services. In 1971, the Employee Assistance Professionals Association was established in the United States to improve EAPs (Employee Assistance Professionals Association, 2014). The main purpose of EAPs is to prevent and resolve any personal or organizational problems that may affect employees’ work performance (Baldino, 1997; Berridge and Cooper, 1994; Colantonio, 1989; Johnston, 1997). Several modern enterprises resolve such problems by identifying high-risk employees and providing them with systematic and professional services to improve work performance.

EAP services can typically be divided into three categories (Ministry of Labor, Executive Yuan, 2017). Those in the first category help employees address problems they encounter in the workplace, those in the second help employees and their family members overcome personal problems, and those in the third help employees maintain and improve their physical and mental health. Regardless of the criteria used to categorize services provided through EAPs, organizations usually aim to provide comprehensive support to their employees through these programs and, in turn, guide them toward healthier physical and mental states. Strong physical and mental health helps employees achieve work–life balance, which is conducive to preventing or minimizing problems that hinder productivity and work performance.

Most studies on EAPs have indicated positive effects of EAPs on employees’ work, life, and health (Chang et al., 2012; Chen, 2012; Kuoppala et al., 2008; Warren and Johnson, 1995), indicating that most EAPs achieve their goals. Those striving to increase the usage and effectiveness of EAPs must consider enterprises’ willingness to provide EAP, the alignment between EAPs and employees’ needs, employees’ awareness of EAPs, employees’ willingness to use EAP, and methods of evaluating and improving EAPs. The negative effects of mentorship, the inability of EAPs to reduce employee stress despite their ability to improve work performance, and the desire of employees with a low risk of obesity to access weight management services have increasingly received attention in research. A detailed review of the results of EAPs and employee feedback may help guide the development and implementation of future EAPs.

### EAP-related factors

This study obtained definitions and examples of EAP measures from the *Employee Assistance Program Promotion Manual*. EAPs were examined in terms of 21 factors across three dimensions (i.e., work, life, and health).

1. *Work dimension*: This dimension encompasses services related to management strategies, adaptation to work, and career assistance.

1. *Employee guidance*: Employee guidance involves the assignment of mentors (i.e., employees with more than 2 years of experience that are familiar with their departments) to new employees and activities, such as on-site observations, dinner parties, and company trips, that enable new employees to quickly adapt to their jobs. The mentorship program is 6 months long, and the employees complete an anonymous questionnaire about their experience at the end of the program. The company then reviews the questionnaire to evaluate the effectiveness of the mentorship program.
2. *Job adaptation*: Measures related to job adaptation often involve satisfaction questionnaires, interviews with employees, and supervisors’ judgment. Human resource personnel, internal counselors, and psychologists formulate customized methods to help employees having trouble adapting to improve and work with supervisors and employees to set performance objectives and schedules. The company then reviews employees’ performance to evaluate their progress, determines whether job changes or adjustments should be made, and ensures that employees continue to receive coaching through the EAP.
3. *Work design*: Measures related to work design often involve assessing the jobs of employees with disabilities, evaluating the appropriateness of their jobs and workload on the basis of their disabilities, and arranging job transfers or retraining. Measures may also involve increasing the accessibility of hardware and physical environments for employees with disabilities, such as offering transportation and audio instructions to employees with visual impairment.
4. *Job changes*: When employees return to work after injury or physical or mental illness, companies often conduct work–health assessments and assign jobs and tasks on the basis of their progress to prevent discouragement due to declines in performance and to ensure their jobs do not jeopardize their health or safety.
5. *Career development*: Measures related to career development may involve career counseling or other forms of career guidance as well as the use of career interest tests to help employees understand themselves from a professional perspective. Supervisors may also work with employees to help them overcome problems and obstacles that they may encounter in the career development process.
6. *Retirement planning*: Companies looking to implement EAP measures related to retirement may form internal support groups, such as older adult–care teams, to help middle-aged and older adult employees explore their values and the meaning of life before retirement and cope with the sense of loss associated with leaving behind a familiar workplace. Companies may use local government resources and provide suggestions to retiring employees to help them plan for life after retirement.
7. *Resignation and job changes*: Measures related to resignation and job changes involve providing opportunities for changes and training, such as the vocational training offered by the Ministry of Labor, to employees who express a desire to resign. They may also involve providing such employees with information regarding stipends for vocational training to ensure they do not encounter financial difficulties after resignation.
8. *Crisis management*: Measures related to crisis management may involve the use of antibullying materials and reporting procedures (which may require company meetings or online platforms, emails, or bulletin boards). They may also involve training sessions, lectures, or classes on workplace bullying prevention, interpersonal communication skills, and employee care skills. Companies may formulate rules for handling bullying and establish complaint-handling committees and internal quick-service windows to cultivate bullying-free workplace environments.

(2) *Life dimension*: This dimension encompasses services designed to help employees solve personal problems that may affect their work.

1. *Financial laws*: Measures related to financial laws may involve assisting employees with their financial planning through tax filing, tax cuts, retirement planning, and debt consolidation to ensure that employees’ work performance is not compromised by factors such as financial instability. Companies may hold seminars on financial planning (e.g., to help employees learn about investment tools and how to reduce their taxes). They may also have internal legal departments or lawyer associations to help employees find lawyers to help them manage common legal matters such as traffic accidents, property and real estate transactions, marriage, and inheritance. The lawyers may provide the employees with additional services related to negotiations and contracts.
2. *Leisure and entertainment*: Measures related to leisure and entertainment may involve building or registering for badminton and table tennis courts, aerobics classes, dance classes, basketball courts, gymnasiums, and swimming pools for employees. Companies may organize events such as family days (on which employees’ families are invited to company activities); employee sports days; employee trips; and celebrations for the three major traditional Chinese festivals, Father’s Day, and Mother’s Day. Such events are often oriented toward having employees’ families visit the companies, test the companies’ products, and become familiar with the employees’ jobs to ensure that they develop positive impressions. Companies may also host parent–child activities such as competitions, pottery making, and concerts.
3. *Marriage and families*: Measures related to marriage and family may involve inviting experts in gender or psychologists to host human lectures or workshops on intimacy and to establish teams to strengthen employees’ communication skills and help them improve their relationships with their partners or spouses. Using their resources and employees’ demographic data, companies may develop services and establish systems to help employees support their families. Managers may submit proposals to revise companies’ leave systems, and companies may add family care leave days or offer flexible work hours.
4. *Childcare and older adult care*: Measures related to childcare and older adult care may involve providing temporary daycare spaces for children or older adults and upgrading childcare services (when companies have their own childcare institutions). Companies may employ qualified childcare workers and offer after-school pick-up and drop-off; after-school tutoring services; talent classes; and winter or summer camps. Companies without the space or budget to build daycare centers and preschools may register with specialized companies and offer discounts on childcare, tuition fees, miscellaneous fees, books, and baby products. They may also provide education subsidies to employees who are single parents.
5. *Interpersonal relationships*: Measures related to interpersonal relationships may involve organizing workplace interpersonal relationship- and communication-related lectures and courses, which may help employees learn how to establish positive relationships with their colleagues and supervisors and prevent workplace bullying. Companies may also host workshops on interpersonal relationships to help employees communicate more effectively with their families and friends in their daily lives.
6. *Insurance planning*: Companies may offer group insurance for employees and their dependents at discount prices. Companies looking to implement measures related to insurance planning may strive to understand employees’ insurance needs and create lists of the companies’ resources (e.g., financial and insurance companies that the companies work with) that employees can reference.
7. *Living assistance*: Measures related to living assistance may involve increasing supervisors' and others’ sensitivity to abnormal work performance and physical and mental signs of domestic violence to protect employees’ privacy and prevent discrimination. Companies may construct hardware environments and implement safety measures, such as security protocols and access control. They may also arrange to have certain employees’ colleagues accompany them home to help minimize the risk of violence. Human resource personnel may assist with relevant referrals and care.

(3) *Health dimension*: This dimension encompasses measures such as the provision of health care through health and medical facilities or services in workplaces to help employees maintain their personal health.

1. *Drug and alcohol cessation*: Measures related to drug and alcohol cessation may involve conducting surveys of employees’ smoking, drug use, and alcohol use and providing information regarding smoking, drug, and alcohol cessation, such as phone numbers for alcohol addiction consultation hotlines and smoking and drug cessation clinics. Companies may also invite employees to participate in support groups.
2. *Worries and anxieties*: Measures related to addressing employees’ worries and anxieties may involve providing psychological counseling services. Such services are designed to address worry and anxiety and to facilitate emotional management. External professionals may explain and administer psychological tests and calculate and expound employees’ test scores. Employees may speak to psychologists one on one to gain a clearer understanding of their test results.
3. *Healthy eating*: Measures related to healthy eating may involve inviting nutritionists to give lectures and consultations on healthy eating and teaching employees how to calculate their caloric intake and control their weight. Companies may label and grade the nutrition and calories in company group meals (e.g., placing red or green labels on high- and low-calorie meals, respectively) and provide maps that indicate the locations of restaurants selling healthy meals near the company office.
4. *Exercise and maintenance*: Measures related to exercise and maintenance may involve providing health information to employees with high body mass index (BMI) recorded during their health checkups and inviting such employees to participate in weight control programs. During these programs, fitness coaches guide employees through aerobic exercises after work or during breaks. Each employee’s BMI is checked again at the end of the program. Companies may also ask executives to commend employees with excellent weight control results, thus inspiring more employees to participate in the programs.
5. *Stress management*: Measures related to stress management may involve using questionnaires to investigate employees' and supervisors’ stress levels and organizing lectures, workshops, and group counseling to address their needs accordingly. Such activities may help employees and supervisors improve their self-awareness and mental health and learn how to properly cope with their stress. Companies may also provide common stress-relieving activities such as massages and yoga. Companies may work with groups with visual impairment to provide stress-relieving massages at fixed locations every month for employees. Companies may also invite teachers to lead yoga classes focused on stretching and relaxation at company offices.
6. *Mental health*: Measures related to mental health may involve recruiting psychologists to work in the company or collaborating with external professional psychological counseling agencies to provide professional, customized psychological counseling services that can help employees manage their personal problems. Companies may use regular promotional meetings or materials to familiarize employees and build their trust. They may also generate monthly, quarterly, or annual reports to evaluate problems and service satisfaction among company employees, which can serve as a reference for adjustment to EAPs and organizational management.

### Stages of organizational development

Early research on organizations typically focused on the static nature of organizations and overlooked aspects related to their dynamic development. This changed in the 1970s with the concept of the organizational life cycle. Proponents of this concept believe that in the process of organizational growth, organizations transition through a series of distinct developmental stages, known as the organizational life cycle stages. In each stage, organizations must manage and overcome different internal and external challenges. Organizational leaders must formulate appropriate strategies to overcome problems, achieve their goals, maintain their competitive edge, and ultimately transition smoothly into the next life cycle stage. If an organization develops incorrectly, it may not survive periods of instability and organizational change (Chen, 2004, 2012; Hanks, 1990). Similarly, the enterprise life cycle theory proposed by Adizes (1989) indicates that enterprises exhibit predictable behavioral patterns that are similar to those of living organisms in their growth and development processes. At different life cycle stages, enterprises face different problems, and their organizational structures, operational strategies, performance, and values change accordingly.

In 1972, Greiner proposed his renowned model of organizational development, in which the organizational development process is divided into five stages: creation, guidance, authorization, coordination, and collaboration. Organizations in the creation stage have informal, simple, flexible, and centralized organizational structures, and the top management of such organizations often adopts a market-oriented management style that emphasizes individualism. As the initially loose organizational structures transition into formal and centralized functional or occupational structures, organizations enter the guidance stage, in which operational efficiency and effectiveness are prioritized. When an organization is in the guidance stage, specialized managers are hired to manage each of the various departments and guide employees in executing management decisions to ensure that the organization can grow through leadership. After entering the standardized authorization stage, most organizations exhibit rapid growth; top management delegates responsibilities to mid-level managers through extensive authorization and assesses the performance of each department through periodic reporting and performance appraisals. As organizations expand, they enter the coordination stage, in which they begin to adopt more formal standards and comprehensive management systems and procedures and sustain their development by coordinating among operational parties. After advancing to the collaboration stage, organizations often used team-based and matrix management systems to resolve complex problems and facilitate innovation. During this stage, top management often adopts a participative management style and avoids intervening in the internal affairs of their organization; the various departments of the organization collaborate through extensive internal negotiations.

To achieve sustainable development, enterprises typically initiate organizational change in response to changes in internal developmental needs or the external environment. When initiating organizational change, enterprises pay particular attention to the union of external relations and internal structures, increases in job positions, replanning and readjustment of work tasks, the retention or dismissal of staff, and increases and reductions in the budget (Mosher, 1967; Sastry, 1997). Organizational change is a type of goal-oriented organizational activity; as they evolve, organizations attempt to achieve their goals by carefully planning and adjusting organizational structures and procedures (Lines, 2005; Robbins, 2003). Lin (2004) noted that when enterprises encounter problems such as outdated working relationships and organizational structures, they often adjust their organizational structure and production efficiency to increase their competitiveness, systematically proceed along the predetermined trajectory of their organizational development, and ultimately achieve their survival and developmental goals. However, when enterprises undergo rapid growth and expansion, an overconfident and rigid organizational culture can easily form because of previous success. Therefore, to maintain performance, the management of an enterprise must formulate operational strategies based on the dynamics of the internal and external environment and adjust the operations of the enterprise accordingly (Adizes, 1979; Porter, 1985; Walker and Ruekert, 1987).

Organizational development is inherently dynamic. Theoretical frameworks, such as enterprise life cycle theory and Greiner’s five-stage model, indicate that the organizational development process comprises a series of distinct stages. An organization’s structure, operational strategies, operational performance, and values vary by life cycle stage. At each stage, organizations must overcome internal and external challenges, such as changes in organizational structure (e.g., from loose to rigid) and management style. To overcome such challenges, organizations must first identify their goals and problems and initiate organizational change or implement operational and management strategies accordingly. When an organization overcomes problems associated with one stage of organizational development, it can successfully proceed to the next stage; if an organization fails to overcome such problems, it may not survive the periods of instability and organizational change associated with transitioning between stages.

## Studies on EAPs and organizational problems

Most studies on EAPs have investigated the effects of EAPs on employees’ attitudes toward work. Similarly, most studies on organizational problems have focused on organizational commitment, performance, and development. This section presents a review of studies on EAPs and organizational commitment, performance, and development.

1. *Studies on EAPs and organizational commitment*
  “Organizational commitment” is an employee’s affective responses to their organization, whereas “job satisfaction” refers to an employee’s affective responses to their work (Williams and Hazer, 1986). Employees’ organizational commitment is affected by factors such as learning opportunities, job satisfaction, the fulfillment of personal needs, access to health care, salaries, benefits, organizational culture, colleagues, job security and stability, and retirement plans (McNeese-Smith and Nazarey, 2001). Employees’ job satisfaction can be used to predict their organizational commitment and can help companies identify potential organizational problems (Seashore and Taber, 1975). Several of these factors relate to the service content of EAPs. EAPs positively affect job satisfaction and organizational commitment. For example, providing channels and measures to help employees communicate, plan their internal careers, and hone their professional skills can increase employees’ job satisfaction. When employees are familiar with and use EAPs, they become more confident in their ability to complete their work tasks, gain a greater sense of self-worth, and feel supported by their organizations. Such feelings tend to manifest as organizational commitment (Li et al., 2016; Liu, 2012; Grant et al., 2008).
  Lee et al. (2008) reported that when organizations provide EAPs that include employee loans, nursing services, stress relief courses, and health benefits, their employees feel at ease in their workplaces and exhibit greater organizational commitment, resulting in lower turnover and strike rates. Lin (2014) noted that supervisors should use stress-related scales to regularly evaluate employees’ work stress and hire psychologists to educate employees on how to cope with stress, which can strengthen employees’ abilities to cope with stress and increase their organizational commitment, resulting in higher retention rates. A study on the implementation of EAPs in Taiwan’s top 500 manufacturing companies revealed that complaint communication services affected employee job satisfaction and that both complaint communication services and consultation services affected employees’ job satisfaction and organizational commitment. Therefore, EAPs can help organizations improve employee job satisfaction and organizational commitment (Tsai, 1998). Currently, Chen et al. (2021) indicated EAPs are negatively correlated with work stress and positively correlated with organizational commitment, job satisfaction, and social support.
2. Studies on EAPs and organizational performances
  EAPs can help companies address employees’ psychological and physical problems, which can help maintain employees’ work stability, increase productivity, and ensure workplace harmony. For example, health services provided through EAPs can improve employees’ moods and daily work performance. Using company exercise facilities more frequently or for longer durations can help employees reduce their work stress and enhance their work performance. The more satisfied employees are with the EAPs available to them, the more their work attitudes and organizational performance improve (Chen, 2013; Sun, 2001; Adam et al., 2007; Lu et al., 2010).
  When companies undergo structural change, EAPs can help stabilize employee morale and the organizational atmosphere. The more satisfied employees are with their EAP services, the more closely they identify with their companies. Therefore, EAPs can serve as strategic management tools that strengthen organizational identity and performance (Lin et al., 2017). Accordingly, companies should view EAPs as welfare systems that ensure satisfaction and happiness among employees and within the organizational culture as a whole. When employees feel happy, they exhibit favorable work performance, creating a win–win situation (Liu, 2012; Lin, 2007).
3. Studies on EAPs and organizational development

The challenges enterprises encounter include global competition and the race for talent (Ministry of Economic Affairs, Executive Yuan, 2018). Appropriately managing and utilizing talent and maintaining competitiveness is key to the sustainable development of an organization. To retain talent and help new talent integrate into the workplace, organizations often use strategies to promote socialization between old and new employees. According to Chou (2008), enterprises should provide comprehensive career planning programs for employees, delegate challenging work tasks to employees to ensure that organizational goals are achieved, and integrate employees’ long-term career planning into their organizational growth plans. These measures can help retain valuable talent and attract new talent, thus facilitating growth and innovation (Chou, 2008). When an organization is newly founded or undergoing rapid growth, the uncertainty associated with developing new technologies is high, and the quality of the organization’s talent becomes the key to its success. Therefore, organizations in either of these stages prioritize educating and training their employees (Wang and Yuan, 2007). As enterprises gradually develop and grow into large-scale enterprises, they start to prioritize the health of their employees to (1) increase employees’ productivity, (2) reduce complaints, (3) facilitate recruitment, and (4) improve their reputation.

According to Chen (2003), the more capital an organization has, the more EAP services it provides to its employees, and most EAP services are related to health. Although EAPs can enhance employees’ work quality and performance, enterprises must have sufficient capital to implement a comprehensive EAP (Chen, 2003). Most enterprises provide their employees with basic services, such as group insurance as well as alcohol, drug, and smoking cessation programs, regardless of their size. Studies have demonstrated that although large-scale enterprises can provide a more comprehensive range of EAP services, small enterprises tend to have higher rates of participation in health promotion schemes (Chang and Lin, 2007; Fielding and Piserchia, 1989; Wilson et al., 1999).

Employees are crucial to the development and growth of organizations. Therefore, organizations attempt to help employees adapt to their organizational cultures by adopting measures and systems designed to ensure organizational commitment and employee retention. In addition, the amount of capital and number of employees an enterprise has are indicators of organizational development; enterprises with greater capital and more employees are generally more capable of implementing EAPs.

## Research design

This study developed a questionnaire based on studies on EAPs and organizational development by using the modified Delphi method. Experts’ experiences and opinions were analyzed to identify the most relevant EAP-related factors in each of the five stages of organizational development. The results can help organizations with resource allocation and in identifying optimal solutions. In the second stage of the study, the fuzzy analytic hierarchy process, proposed by Saaty (1980), was used to determine the weights of the key factors for EAP implementation in each stage of organizational development.

### Study sample

Human resources specialists with experience formulating and planning EAPs in Taiwan were recruited for expert panels. Each expert completed a modified Delphi questionnaire and fuzzy analytic hierarchy process questionnaire. Delbecq et al. (1975) indicated that when the heterogeneity among members of an expert panel is high, the size of the Delphi expert panel can be as small as 5–10 members; however, when the homogeneity among the members is high, the size of the panel must be expanded to 15–30 members. This rule cannot be generalized to all fields because the sizes of the parent populations vary. Dalkey (1969) indicated that generally, the lowest rates of population error and highest levels of validity can be achieved when a Delphi expert panel with more than 10 members is used. In this study, the same experts who completed the modified Delphi questionnaire were invited to complete the fuzzy analytic hierarchy process questionnaire. The sole difference between the fuzzy and conventional analytic hierarchy processes is that the fuzzy analytic hierarchy process is based on fuzzy theory. In accordance with the guidelines established in another study (Saaty, 1980), the size of the expert panel in the present study was 5–15 experts.

The participants had experience with at least three stages of organizational development. Purposive sampling was used to recruit 13 experts for the questionnaire surveys. Most were women (92.3%), held a graduate degree or above (84.62%), and had 11–20 (45.16%) or at least 21 (45.16%) years of experience. Most were managers (38.46%), and some were deputy managers (15.38%) and management specialists (15.38%). A total of 46.15% of the experts worked in organizations with 250 or fewer employees, and 30.77% worked in organizations with 251–500 employees. All experts had experience implementing EAPs; 38.46%, 30.77%, and 30.77% had implemented 10 or more, 6–10, and 1–5 EAPs, respectively (Table 1).

**Table 1:** Demographics of experts who completed the modified Delphi and fuzzy analytic hierarchy process questionnaires (*N* = 13).

Item	Variable	Frequency	Percentage
Sex	Man	1	7.69
	Woman	12	92.30
Educational attainment	College (junior college)	2	15.38
	Graduate school or above	11	84.62
Seniority	10 years or fewer	1	7.69
	11–20 years	6	46.15
	21 years or more	6	46.15
Current title	Assistant professor	1	7.69
	Division Chief	1	7.69
	Administrator	2	15.38
	Director	1	7.69
	Assistant Manager	2	15.38
	Manager	5	38.46
	Human resources director	1	7.69
Current organization size	250 employees or fewer	6	46.15
	251–500 employees	4	30.77
	501–1000 employees	2	15.38
	1001–1500 employees	0	0.00
	1501 employees or more	1	7.69
Experience in implementing EAPs	Yes	13	100.00
Number of times implementing an EAP	1–5 times	4	30.77
	6–10 times	4	30.77
	>10 times	5	38.46

### Research tools

#### Modified Delphi questionnaire

A structured questionnaire was used to investigate the EAP measures implemented by organizations at various stages of organizational development. The questionnaire items and answers were developed with reference to the literature. By referencing the *Employee Assistance Program Promotion Manual* published by Taiwan’s Ministry of Labor in 2017, three major EAP dimensions, namely the work, living, and health dimensions, were identified. A total of 21 factors across the three dimensions were used for analysis. Greiner’s five-stage model of organizational development was used as the theoretical framework of the organizational development process. When completing the questionnaire, the experts were required to assess the importance of each factor at each stage of organizational development on the basis of their experience and professional opinions. Each item on the questionnaire was rated on a 5-point Likert scale ranging from 1 (*strongly disagree*) to 5 (*strongly agree*).

The modified Delphi method involves using interquartile ranges as tools to determine consistency among experts’ answers. An interquartile range is half the distance of the 50% opinion distribution, and the lower the range is, the more concentrated the expert opinions are. According to Faherty (1979), an interquartile range of ≤0.60 indicates a high degree of agreement, whereas an interquartile range of 0.60–1.00 indicates a moderate degree of agreement. The experts’ responses on the modified Delphi questionnaires were tested using interquartile ranges. After the questionnaires were collected, Microsoft Excel was used to calculate the interquartile range for each item, and the items with corresponding interquartile ranges of ≤0.60 were included in the fuzzy analytic hierarchy process questionnaires.

#### Fuzzy analytic hierarchy process questionnaire

The fuzzy analytic hierarchy process questionnaire was based on the analysis of the survey conducted using the modified Delphi questionnaire in the first stage of the study. It was divided into three sections. The first and second sections consisted of paired comparisons of the three EAP dimensions and of the factors in each of the three dimensions, respectively. In these two sections, items related to organizational development processes were incorporated as situational discussion items. The third section was used to collect the experts’ personal demographic information. The questionnaire was designed to determine the EAP-related measures related to work, life, and health that the Delphi experts deemed crucial for organizations in the creation, guidance, authorization, coordination, and collaboration stages.

The fuzzy analytic hierarchy process questionnaires were analyzed through the following procedure.

1. A data consistency index of ≤0.1 indicated that the answers were consistent and could be used to construct pairwise comparison matrices and calculate weights.

2. A Microsoft Excel spreadsheet was used to calculate the geometric means of the valid data to the third decimal place and construct the corresponding pairwise comparison matrices.

3. The Super Decisions software package was used to make pairwise comparisons between the EAP factors at every level and rank their importance, and the data were compiled into Excel spreadsheets.

## Data analysis

### Analysis of the modified Delphi questionnaire responses

The numbers of EAP measures implemented in each of the five stages of organizational development are as follows: 10 in the creation stage, 14 in the guidance stage, 16 in the authorization stage, and 18 each in the coordination and collaboration stages. These results indicate that as organizations develop, the number of EAP measures they implement tends to increase. Thus, as organizations gradually approach the collaboration stage from the creation stage, the range of EAP measures they implement widens. Although the numbers of EAP measures implemented in the coordination and collaboration stages were identical, the number of EAP measures corresponding to the work and health dimensions in each stage differed considerably. Compared with the collaboration stage, an additional category of EAP measures in the work dimension, namely position changes, was implemented in the coordination stage. Compared with the coordination stage, an additional category of EAP measures corresponding to the health dimension, namely those designed to address employees’ worries and anxiety, was implemented in the collaboration stage. This indicates that when organizations transition from managing departments separately to implementing team-based management strategies, their prioritization of work- and health-related assistance changes accordingly. The number of EAP measures corresponding to the work dimension did not increase linearly as organizations progressed through the stages. More EAP measures corresponding to the work dimension were implemented in the guidance stage than in the authorization stage and in the coordination stage than in the collaboration stage. Thus, the changes in work-related EAP measures differed from those in the life- and health-related measures, as well as that in the EAP, measures overall.

Although the EAP services provided by each enterprise differed somewhat at each stage of development, some were provided by all enterprises: services related to work design in the work dimension; services related to family and marriage, interpersonal relationships, and life in the life dimension; and services related to dietary health in the health dimension. Therefore, enterprises focus on (1) employees with physical or mental disabilities having access to accommodations and relevant evaluations, (2) employees’ ability to juggle their workload with family life after marriage, (3) employees’ ability to interact and communicate with others inside and outside the workplace, (4) employees being subject to domestic violence, and (5) employees’ ability to control their caloric intake for diet and weight control. Three, one, and one of these focuses can be addressed through EAP services in the life, work, and health dimensions, respectively. This distribution ratio matched the ratio of implemented EAP services in each dimension in most of the stages of organizational development, with the exception of the guidance stage, in which more EAP services corresponding to the work and health dimensions are implemented Table 2.

**Table 2:** Results of modified Delphi questionnaire survey.

		Organizational development stages				
		Creation	Guidance	Authorization	Coordination	Collaboration
EAP measure dimensions	Work	3	5	4	6	5
	Living	4	4	7	7	7
	Health	3	5	5	5	6
	Total	10	14	16	18	18

### Analysis of the responses to the fuzzy analytic hierarchy process questionnaire

On the basis of the collective feedback from the expert panel, the EAP measures most frequently implemented in each of the five stages of organizational development were identified. Weighted pairwise comparisons were performed on the basis of the experts’ suggestions regarding the importance of each factor. The weights and orders of the factors are presented in Table 3.

**Table 3:** Weights of factors corresponding to EAP measures at each stage of organizational development.

Organizational development stage	Creation	Guidance	Authorization	Coordination	Collaboration
A. Criteria (weight/ranking) a Factor (weight/ranking)	A. Work dimension (0.72/1) a1 Work design (0.75/1) a2 Position change (0.17/2) a3 Retirement planning (0.08/3)	A. Work dimension (0.70/1) a1 Employee guidance (0.25/2) a2 Work adaptation (0.26/1) a3 Work design (0.23/3) a4 Position change (0.17/4) a5 Career development (0.10/5)	A. Work dimension (0.66/1) a1 Employee guidance (0.23/2) a2 Work adaptation (0.14/4) a3 Work design (0.44/1) a4 Career development (0.19/3)	A. Work dimension (0.45/1) a1 Work adaptation (0.28/1) a2 Work design (0.12/5) a3 Position change (0.19/2) a4 Retirement planning (0.17/3) a5 Resignation and career change (0.08/6) a6 Crisis management (0.16/4)	A. Work dimension (0.43/1) a1 Work adaptation (0.15/5) a2 Work design (0.20/3) a3 Retirement planning (0.26/1) a4 Resignation and career change (0.17/4) a5 Crisis management (0.23/2)
B. Criteria (weight/ranking) b Factor (weight/ranking)	B. Living dimension (0.11/3) b1 Family and marriage (0.23/3) b2 Interpersonal relationships (0.39/1) b3 Insurance planning (0.24/2) b4 Life assistance (0.14/4)	B. Living dimension (0.11/3) b1 Family and marriage (0.27/2) b2 Care for dependent children and older adults (0.15/4) b3 Interpersonal relationships (0.43/1) b4 Life assistance (0.15/3)	B. Living dimension (0.13/3) b1 Financial and legal support (0.12/5) b2 Recreation and entertainment (0.15/3) b3 Family and marriage (0.11/6) b4 Care for children and older adults (0.15/4) b5 Interpersonal relationships (0.23/1) b6 Insurance planning (0.16/2) b7 Life assistance (0.09/7)	B. Living dimension (0.18/3) b1 Financial and legal support (0.07/7) b2 Recreation and entertainment (0.12/5) b3 Family and marriage (0.11/6) b4 Care for children and older adults (0.16/2) b5 Interpersonal relationships (0.23/1) b6 Insurance planning (0.16/3) b7 Life assistance (0.14/4)	B. Living dimension (0.20/3) b1 Financial and legal support (0.21/1) b2 Recreation and entertainment (0.11/6) b3 Family and marriage (0.15/4) b4 Care for children and older adults (0.16/3) b5 Interpersonal relationships (0.17/2) b6 Insurance planning (0.12/5) b7 Life assistance (0.08/7)
C. Criteria (weight/ranking) c Factor (weight/ranking)	C. Health dimension (0.18/2) c1 Worries and anxiety (0.23/3) c2 Healthy diet (0.33/2) c3 Mental health (0.44/1)	C. Health dimension (0.19/2) c1 Worries and anxiety (0.27/2) c2 Healthy diet (0.15/4) c3 exercise and body maintenance (0.17/3) c4 Stress management (0.30/1) c5 Mental health (0.12/5)	C. Health dimension (0.22/2) c1 Drugs and alcohol cessation (0.22/2) c2 Worries and anxiety (0.21/3) c3 Healthy diet (0.14/5) c4 exercise and body maintenance (0.19/4) c5 Stress management (0.24/1)	C. Health dimension (0.37/2) c1 Drugs and alcohol cessation (0.41/1) c2 Healthy diet (0.13/4) c3 exercise and body maintenance (0.12/5) c4 Stress management (0.19/2) c5 Mental health (0.15/3)	C. Health dimension (0.37/2) c1 Drugs and alcohol cessation (0.28/1) c2 Worries and anxiety (0.13/4) c3 Healthy diet (0.11/6) c4 exercise and body maintenance (0.13/5) c5 Stress management (0.22/2) c6 Mental health (0.14/3)

In the creation stage, organizations prioritized work-related EAP measures. The weight of the work dimension was 0.72, whereas those of the health and life dimensions were 0.18 and 0.11, respectively. The consistency ratios (CRs) for the work, life, and health dimensions were 0.06, 0.02, and 0.01, respectively, all of which were ≤0.10, indicating that the judgment of the Delphi experts was consistent throughout the evaluation. Among the work-related EAP measures implemented by organizations in the creation stage, those related to work design were most crucial, as evidenced by the relative weight of the work design factor (0.75) being much higher than those of the other two factors in the work dimension (namely position changes [0.17] and retirement planning [0.08]). In the life dimension, the most crucial factor (in terms of relative weight) was interpersonal relationships (0.39), followed by insurance planning (0.24), family and marriage (0.23), and life assistance (0.14). In the health dimension, the most crucial factor was mental health (0.44), followed by dietary health (0.33) and worries and anxiety (0.23).

Similar to organizations in the creation stage, organizations in the guidance stage highly prioritized EAP measures in the work dimension. For organizations in the guidance stage, the relative weight of the work dimension was 0.70, whereas those of the health and living dimensions were 0.19 and 0.11, respectively. The CRs of the work, living, and health dimensions were 0.00, 0.02, and 0.05, respectively, all of which were ≤0.10, indicating that the judgments of the Delphi experts remained consistent throughout the evaluation process. For organizations in the guidance stage, the most crucial work-related EAP measure (in terms of relative weight) was work adaptation (0.26), followed by employee guidance (0.25), work design (0.23), position changes (0.17), and career development (0.10). In the living dimension, the most crucial factor was interpersonal relationships (0.43), followed by family and marriage (0.27), life assistance (0.15), and care for dependent children and older adults (0.15). In the health dimension, the most crucial factor was stress management (0.30), followed by worries and anxiety (0.27), exercise and body maintenance (0.17), dietary health (0.15), and mental health (0.12).

Organizations in the authorization stage also prioritized EAP measures in the work dimension. In the authorization stage, the relative weight of the work dimension was 0.66, whereas those of the health and life dimensions were 0.22 and 0.13, respectively. The CRs of the work, life, and health dimensions were 0.03, 0.04, and 0.03, respectively, all of which were ≤0.10, indicating that the judgment of the Delphi experts was consistent throughout the evaluation. In the authorization stage, the most crucial work-related EAP factor (in terms of relative weight) was work design (0.44), followed by employee guidance (0.23), career development (0.19), and work adaptation (0.14). In the life dimension, the most crucial factor was interpersonal relationships (0.23), followed by insurance planning (0.16), recreation and entertainment (0.15), care for dependent children and older adults (0.15), financial and legal support (0.12), family and marriage (0.11), and life assistance (0.09). In the health dimension, the most crucial factor was stress management (0.24), followed by drug and alcohol cessation (0.22), worries and anxiety (0.21), exercise and body maintenance (0.19), and dietary health (0.14).

Organizations in the coordination stage also prioritized EAP measures in the work dimension. For organizations in the coordination stage, the relative weight of the work dimension was 0.66, which was much lower than those for organizations in the creation, guidance, and authorization stages. For organizations in the coordination stage, the relative weight of the health dimension was 0.37, which was much higher than those for organizations in the creation, guidance, and authorization stages. The relative weight of the life dimension for organizations in the coordination stage was 0.18. The CRs of the work, life, and health dimensions were 0.05, 0.03, and 0.02, respectively, all of which were ≤0.10, indicating that the judgment of the Delphi experts was consistent throughout the evaluation. For organizations in the coordination stage, the most crucial factor (in terms of relative weight) was work adaptation (0.28), followed by position changes (0.19), retirement planning (0.17), crisis management (0.16), work design (0.12), and resignation and career change (0.08). In the life dimension, the most crucial factor was interpersonal relationships (0.23), followed by care for dependent children and older adults (0.16), insurance planning (0.16), life assistance (0.14), recreation and entertainment (0.12), family and marriage (0.11), and financial and legal support (0.07). In the health dimension, the most crucial EAP measures were those related to drug and alcohol cessation (0.41), followed by those related to stress management (0.19), mental health (0.15), dietary health (0.13), and exercise and body maintenance (0.12).

The priority levels of the three EAP dimensions for organizations in the collaboration stage were similar to that for organizations in the coordination stage. The relative weight of the work dimension for organizations in the collaboration stage was 0.43, which was much lower than those for organizations in the creation, guidance, and authorization stages. The relative weight of the health dimension was 0.37, which was much higher than those for organizations in the creation, guidance, and authorization stages. The relative weight of the life dimension was 0.20. The CRs of the work, life, and health dimensions were 0.019, 0.03, and 0.01, respectively, all of which were ≤0.10, indicating that the judgment of the Delphi experts was consistent throughout the evaluation. The analysis revealed that for organizations in the collaboration stage, the relative importance of factors corresponding to the work, life, and health dimensions does not differ much. The work-related factors, in descending order of relative weights, were as follows: retirement planning (0.26), crisis management (0.23), work design (0.20), resignation and career change (0.17), and work adaptation (0.15). The life-related factors, in descending order of relative weights, were as follows: financial and legal support (0.21), interpersonal relationships (0.17), care for dependent children and older adults (0.16), family and marriage (0.15), insurance planning (0.12), recreation and entertainment (0.11), and life assistance (0.08). The health-related factors, in descending order of relative weight, were as follows: drug and alcohol cessation (0.28), stress management (0.22), mental health (0.14), worries and anxiety (0.13), exercise and body maintenance (0.13), and dietary health (0.11).

## Conclusions

The creation stage is the initial stage of organizational development. In this stage, an enterprise usually has an informal organizational structure, and senior managers tend to adopt individualistic management styles and to value market performance. Therefore, enterprises in the creation stage tend to adopt EAP measures corresponding to the work dimension (0.718), with a particular focus on maintaining or improving employee work performance. EAP measures related to work design (0.751) and position changes (0.169) involve evaluations of employees’ responsibilities performed to adjust employees’ duties and help employees maintain or increase their productivity. Measures related to work design, position changes, and retirement planning (0.080) are designed to address problems encountered by employees with special needs. Organizations often lack resources for employees in the early stages of development and therefore tend to prioritize assisting employees with special needs. The same applies to measures related to mental health (0.441) and worries and anxiety (0.226) in the health dimension (0.176); both are only provided to employees in need by professionals or departments.

In the guidance stage, organizations assist all employees and help them care for or obtain professional care for family members by implementing measures related to care for children or older adults (0.146). These measures, in addition to measures related to family and marriage (0.272) and life assistance (0.152), help employees stabilize their families, reduce family-related stress, and focus on their work. As an organization enters the guidance stage, both the coverage and content of its EAP services increase. In addition, the number of department leaders between employees and senior managers increases, whereas the number of employees directly managed by each department leader is lower than that managed by senior managers. This structural change creates opportunities to identify difficulties employees encounter in the workplace and provide assistance. Organizations entering the guidance stage also begin to value business performance and are more willing to use their resources to help employees reduce stress unrelated to work.

In the authorization stage, companies still implement the life-related measures adopted in the creation and guidance stages. At this stage, measures related to financial and legal support (0.118) and recreation and entertainment (0.146) are first incorporated into a company’s EAPs. Measures related to recreation and entertainment can enable employees’ family members to learn about their organizations, thus increasing the degree to which they identify with the organization as well as their support for employees’ work. Accordingly, an organization in the authorization stage utilizes and refines life-related EAP measures they may have adopted in the creation and guidance stages and expands their EAPs to include employees’ families.

As an organization enters the coordination stage, job responsibilities, management systems, and operating procedures in each department become increasingly standardized. More coordination is, therefore, necessary to ensure the organization’s continuous growth. Among the five stages of organizational development, the coordination stage involves the implementation of the most comprehensive range of work-related measures; these measures mainly involve designating job responsibilities, confirming employee placement, and determining whether to dismiss employees. These measures include those related to work adaptation (0.279), position changes (0.193), work design (0.117), retirement planning (0.173), and resignation and career change (0.081). EAPs may resolve the developmental challenges faced by organizations in this stage, such as excessive management layers, prolonged decision-making cycles, and staff redundancy. Although most measures implemented during the coordination stage correspond to the work dimension, the relative importance of the work dimension (0.454) significantly decreases during this stage. This is likely because, by this stage, most organizations have already implemented numerous work-related measures and must rely on employee cohesion to sustain their large structures and prevent a decline in corporate cohesion. Consequently, they may adopt measures related to health (0.366) and life (0.180) to help employees feel cared for and valued, which enhances team and corporate cohesion.

Complexity and rigidity are inevitable problems for organizations during the development process. As organizations enter the collaboration stage, they strive to resolve these problems, innovate through team and matrix management, and begin to stabilize. Therefore, the order of priority among the work (0.434), life (0.196), and health (0.370) dimensions remains roughly the same as that in the coordination and collaboration stages. By the time an organization reaches the collaboration stage, it is relatively old, and its employees may begin to retire. Therefore, EAP measures related to retirement planning (0.258), including those designed to help employees arrange for life after retirement or adapt to the sense of loss arising from leaving the workplace, may start to receive more attention. Because organizations in the collaboration stage tend to be large and often have comprehensive systems, the establishment and cultivation of friendly workplace culture are crucial, and additional measures related to crisis management (0.229) are used to address workplace bullying.

## Discussion and suggestions

### Discussion

According to the *Employee Assistance Program Promotion Manual* published by Taiwan’s Ministry of Labor in 2017, when an organization implements an EAP, it tends to prioritize EAP measures corresponding to the work dimension over those corresponding to the life and health dimensions. When organizations are first founded, they are often unable to implement a comprehensive range of EAP measures because of their limited resources and capacities. Therefore, during the creation stage, they focus their attention primarily on problems that directly affect employees’ work performance. Such organizations may therefore be inadequately equipped to help employees with problems related to their living situations or physical and mental health. These results are consistent with those reported by Arthur (2000) and Chen (2003). Measures related to work design were identified as the most crucial among all the EAP measures corresponding to the work dimension, which may be explained by the results reported by Wu et al. (2003) and Chang (2014), which indicate that when organizations help employees with physical disabilities resolve software- and hardware-related problems in the workplace, the employees’ work performance substantially improves. In addition, some researchers have noted that regardless of size, most enterprises provide alcohol, smoking, and drug cessation services as well as group insurance services to their employees (Chang and Lin, 2007; Fielding and Piserchia, 1989). However, the organizations in the present study did not provide alcohol, smoking, and drug cessation services during the creation stage. This may be because the organizations were afraid of violating employees’ privacy and wanted to avoid the increased labor and time costs associated with providing cessation services and investigating employees’ drinking and smoking habits.

This study revealed that the priority levels of the three EAP dimensions for organizations in the guidance stage were identical to those for organizations in the creation stage. The organizations first offered EAP services related to work adaptation, employee guidance, exercise and body maintenance, and stress management in the guidance stage. This indicates that in the guidance stage, organizations often begin to pay attention to their employees’ competitiveness and are therefore more willing to invest resources into the care, management, and effective mobilization of employees to improve organizational commitment, performance and efficiency of operations. These findings are consistent with those of Lee et al. (2008). Some researchers have noted that smaller enterprises often encounter difficulties in providing services related to workplace health, which may be because they lack professional knowledge and skills (Chen, 2005). The results of this study are partially consistent with this because, in the guidance stage, the priority levels of the EAP measures corresponding to the life and health dimensions were much lower than those of the EAP measures corresponding to the work dimension. However, some of the results are inconsistent with those of Chen (2005); the organizations in the guidance stage were only four measures away from providing the full range of EAP services corresponding to the life and health dimensions. A possible explanation for this is that smaller enterprises employ single-service or resource-linkage models in providing EAP services corresponding to the living and health dimensions and can therefore provide EAP services focusing on various topics (Ministry of Labor, Executive Yuan, 2017).

During the authorization stage, companies often incorporate measures related to financial and legal support, recreation and entertainment, and insurance planning into their EAPs, making the authorization stage the first developmental stage in which organizations provide the full range of EAP services corresponding to the life dimension. Among the EAP measures corresponding to the life dimension, those related to interpersonal relationships, insurance planning, and recreation and entertainment are most frequently prioritized. The authorization stage is the first stage in which the organizations usually implement EAP measures related to drug and alcohol cessation (corresponding to the health dimension). These results indicate that organizations in the authorization stage are better equipped—both in terms of resources and professional skills—to provide EAP services corresponding to multiple dimensions. In addition, these results indicate that during the authorization stage, organizations begin to realize the importance of family-friendly systems and policies in helping employees achieve a work–life balance, which may be why many organizations start to provide EAP services after reaching the authorization stage. These results are consistent with those reported by Fielding and Piserchia (1989) and Warren and Johnson (1995). Despite the substantial increases in the priority levels and numbers of life- and health-related EAP measures, work-related EAP measures were still the top priority for companies in the authorization stage. In this stage, measures related to career development first emerged as one of the top three categories of EAP measures corresponding to the work dimension. These results indicate that as organizations develop, they begin to pay more attention to their employees’ career development by shifting their attention away from short-term work-related problems. These results also indicate that EAP measures related to career development facilitate the growth and development of both employees and organizations, which is consistent with the findings of Chou (2008).

Similarly to organizations in the authorization stage, organizations in the coordination stage often provide the full range of EAP services corresponding to the life dimension. In the coordination stage, the priority levels of the living-related EAP measures increased, although they still lagged behind those of the work- and health-related EAP measures. Work-related EAP measures remained the most crucial EAP measures for companies in the coordination stage, but their priority levels in the authorization stage were lower than they were in previous stages. The coordination stage is also often the first stage in which organizations start to provide the full range of EAP services corresponding to the work dimension. The priority level of EAP measures corresponding to the health dimension increases substantially in this stage, indicating that as organizations grow and develop, they become capable of providing an increasingly diverse range of EAP services. The increases and decreases in the priority levels of the three EAP dimensions suggest that although organizations continue to prioritize work-related EAP measures over time, they also start to pay more attention to problems related to the life and health dimensions. These results are consistent with those reported by Chen (2003, 2005), and Yu (2007). In 1975, Seashore and Taber revealed that job satisfaction can be used to predict employees’ organizational commitment and identify problems in an organization. In the coordination stage, organizations often attempt to address with developmental crises, such as an excessively hierarchical management structure, redundant personnel, an increasing organization size, and increasing organizational divisiveness, by implementing EAPs. To this end, organizations in the coordination stage often prioritize the implementation of EAP measures that have been determined to enhance employees’ job satisfaction and organizational commitment (Li et al., 2016; Liu, 2012; McNeese-Smith and Nazarey, 2001; Pettigrew, 1988; Turnage and Spielberger, 1991). These measures include those related to work adaptation, position changes, retirement planning, interpersonal relationships, care for dependent children and older adults, insurance planning, stress management, and mental health.

Organizations in the collaboration stage have typically grown to a certain size and possess a substantial number of resources. Because they usually have a sufficient number of resources and amount of capital, they are more willing to invest in workplace health. Organizations in the collaboration stage are more capable of providing a comprehensive range of EAP measures corresponding to the life and health dimensions than are those in other developmental stages. Although the work dimension remained the most frequently prioritized, followed by the health and life dimensions, the difference in the priority levels of the work and health dimensions was minimal, and the number of implemented EAP measures corresponding to the health dimension was slightly higher than that of the measures corresponding to the work dimension. These results are consistent with those reported by Chen (2005) and Chen (2003). The results suggest that in the collaboration stage, EAP measures related to interpersonal relationships remain among the most frequently prioritized measures corresponding to the life dimension and that measures related to crisis management first emerged as one of the top three most crucial categories of EAP measures corresponding to the work dimension. These results indicate that in the collaboration stage, organizations begin to pay attention to workplace bullying and its potential negative effects on employees’ productivity and job satisfaction. To address workplace bullying, organizations often attempt to help their employees create and maintain strong interpersonal relationships through campaigns, seminars, and channels for reporting workplace bullying. These findings are similar to those reported by Tsai (1998) and Hung (2008). Although organizations in the collaboration stage implemented the same number of EAP measures as did those in the coordination stage in the present study, they differed in terms of their purpose for providing EAP services. Data on the differences among the priority levels of the three EAP dimensions and the distribution of implemented measures across the dimensions indicate that organizations in the collaboration stage mainly provide EAP services to ensure employee satisfaction and well-being and organizational development. They did not consider the cost of EAP services to be burdensome. These results are consistent with those of Lin (2007) and Liu (2012).

The Taiwanese government has promoted work–life balance and established policies and awards to encourage companies to implement EAPs. The Ministry of Labor of the Executive Yuan released the latest version of the *Employee Assistance Program Promotion Manual* in 2017; the manual categorizes EAP services into the work, life, and health dimensions, all of which have corresponding EAP measures. However, the manual does not describe the implementation of each measure. The EAP implementation cases presented in the manual differ from these assistance measures, which may have prevented readers from fully understanding the meaning of each measure. This study summarized the EAP implementation cases in the manual and the meaning of each EAP measure. This study used the modified Delphi method to determine the EAP measures most frequently used by organizations in the creation, guidance, authorization, coordination, and collaboration stages. In addition, the manual does not address the implementation of measures corresponding to the life dimension. Compared with the measures described in the manual, the EAP measures described herein are more detailed in content. This study may therefore serve as a reference for human resources personnel in planning EAPs and for companies in selecting appropriate EAP measures for each developmental stage.

This study also used the fuzzy analytic hierarchy process to assess the relative importance of each EAP dimension and measure in each of the stages. The results may serve as a reference for human resources personnel planning and implementing EAPs. Given that an organization’s resources are exhaustible, EAPs must be carefully planned and evaluated. The results also indicate that the relative weight of each EAP dimension or measure can be used to determine the resources that organizations should invest in each dimension or measure. When the weight of an EAP measure is high, organizations tend to prioritize (and invest more in) services related to that measure.

### Suggestions

The *Employee Assistance Program Promotion Manual* classifies EAP service models as internal, external, integrated, resource-linkage, or single-service models on the basis of differences in their approaches to problem identification, evaluation, and resolution. Combining this EAP service model categorization system with the results of the present study revealed that organizations adopt different EAP service models in each developmental stage by considering factors such as resource availability, organizational structure, and current EAP measures.

(i) In the creation stage, organizations often have a limited number of resources and informal organizational structures. They usually do not have any department personnel in charge of implementing EAPs. Employees who have specific needs can utilize free social resources provided by their organizations. Therefore, organizations in the creation stage should adopt the resource-linkage EAP service model, which is the most labor- and cost-efficient model for organizations in this stage.
(ii) In the guidance stage, organizations seek to increase their operational efficiency and are therefore more willing to provide additional EAP services to help employees maintain or increase their productivity. However, because organizations in the guidance stage still have a limited number of resources, they may benefit from adopting the single-service EAP service model, which is low-cost and can be used to address problems.
(iii) In the authorization stage, organizations often have more resources, enabling them to provide a range of EAP services. Organizations in this stage tend to emphasize family-friendly policies and attempt to expand their EAPs to their employees’ family members. Organizations in the authorization stage should adopt the external EAP service model, which involves a range of services and protecting employees’ privacy.
(iv) In the coordination stage, organizations attempt to resolve developmental crises by implementing EAPs. The personnel in charge of planning and implementing EAPs must understand the internal structures of their organizations and design EAPs accordingly. Organizations in the coordination stage have more resources to invest in EAPs than do those in the authorization stage. Therefore, they should adopt the internal EAP service model, which requires the service provider to be familiar with the organizational culture and employees’ needs.
(v) Organizations in the collaboration stage possess a sufficient number of resources, can provide a range of EAP services, and prioritize all three EAP dimensions. Therefore, internal personnel familiar with the organization and implementing EAPs and external organizations with expertise in EAPs should plan and implement EAPS to ensure that they cover all three dimensions. Organizations in the collaboration stage should adopt the integrated EAP service model, which is associated with a range of services and service channels.

Selecting the appropriate EAP service model for each stage can help organizations optimize resource allocation. An effective EAP can also help attract outstanding talent. Selecting the appropriate service model enables those in charge of EAPs to efficiently delegate tasks and responsibilities and can facilitate planning and implementation. EAPs can help employees resolve their problems and remind them of their organizations’ efforts to help.

Because EAPs are strategic management tools designed to enhance employees’ organizational identification and the organizations’ performance, an EAP evaluation process should be developed. Careful evaluation can prevent an EAP from becoming a mere formality. The results of this study can be used in conjunction with the Plan, Do, Check, and Action (PDCA) management cycle framework to design an effective EAP evaluation process. When an EAP is in the “Plan” stage, the main task is to identify the guiding principles and goals. The relative weights of EAP dimensions and measures in each organizational stage (reported herein) may serve as a reference for determining the guiding principles and goals of a given EAP. In the “Do” stage, the main objective is to realize the content of the EAP plan. The descriptions of each EAP measure herein may serve as a reference in the content design process. After the plan is executed, the “Check” process (which involves confirmation, supervision, and assessment) begins. Problems with the design of an EAP are identified through the evaluation of differences between results and expectations. The findings of this study regarding the relative importance of each EAP measure in each stage of organizational development can be used to facilitate assessment and problem identification. Finally, in the “Action” stage, the assessment results are analyzed, and the EAP is modified accordingly. Employee feedback and suggestions are considered during the planning of EAPs. Integrating the results of this study into the PDCA management cycle framework can enable organizations to maximize the effects of EAPs through improvement and adjustment.

### Limitations

Because of constraints related to time and human resources, experts serving in organizations that had undergone all five stages of organizational development could not be recruited for the questionnaire survey. Instead, each expert was asked to complete the questionnaire for all five stages of organizational development. To minimize the effect of this limitation, human resources personnel who had experience implementing EAPs were recruited for the expert panel. Many of these experts had participated in at least 10 EAPs (38.46%), and most had at least 11 years of experience (92.3%).

## Supplementary information

Appendices

## Data Availability

The datasets generated during the current study are not publicly available but are available from the corresponding author on reasonable request.
