# Supplementary material for: Stages of organizational development and employee assistance programs in Taiwan
Source: Humanit Soc Sci Commun. 2023 Mar 7;10(1):87. doi: 10.1057/s41599-023-01567-4 (PMC9990578; doi:10.1057/s41599-023-01567-4)
Supplement: Supplementary file 1 — Appendices [file 41599_2023_1567_MOESM1_ESM.docx]

Appendix 1. Part of Modified Delphi Method Questionnaire

**First part EAP measures of employee guidance**

| Employee guidance involves the assignment of mentors (i.e., employees with more than 2 years of experience that are familiar with their departments) to new employees and activities, such as on-site observations, dinner parties, and company trips, that enable new employees to quickly adapt to their jobs. The mentorship program is 6 months long, and the employees complete an anonymous questionnaire about their experience at the end of the program. The company then reviews the questionnaire to evaluate the effectiveness of the mentorship program. | Extremely agree | agree | No opinion | disagree | Extremely  disagree |
| --- | --- | --- | --- | --- | --- |
| 1. The EAP measures of employee guidance above can be implemented in the Creation Stage. | □ | □ | □ | □ | □ |
| 2. The EAP measures of employee guidance above can be implemented in the Guidance Stage. | □ | □ | □ | □ | □ |
| 3. The EAP measures of employee guidance above can be implemented in the Authorization Stage. | □ | □ | □ | □ | □ |
| 4. The EAP measures of employee guidance above can be implemented in the Coordination Stage. | □ | □ | □ | □ | □ |
| 5. The EAP measures of employee guidance above can be implemented in the Collaboration Stage. | □ | □ | □ | □ | □ |

Appendix 2. Part of Fuzzy Analytic Hierarchy Process Method Questionnaire

Q1: Please tick the relatively importance of each EAP dimensions in the “Creation Stage”.

| Intensity  Dimension | Absolutely important |  | Extremely  important |  | Quite important |  | Slightly important |  | Equally important |  | Slightly important |  | Quite important |  | Extremely  important |  | Absolutely important | Dimension |
| --- | --- | --- | --- | --- | --- | --- | --- | --- | --- | --- | --- | --- | --- | --- | --- | --- | --- | --- |
|  | 9 | 8 | 7 | 6 | 5 | 4 | 3 | 2 | 1 | 2 | 3 | 4 | 5 | 6 | 7 | 8 | 9 |  |
| Work dimension | □ | □ | □ | □ | □ | □ | □ | □ | □ | □ | □ | □ | □ | □ | □ | □ | □ | Living dimension |
| Work dimension | □ | □ | □ | □ | □ | □ | □ | □ | □ | □ | □ | □ | □ | □ | □ | □ | □ | Health dimension |
| Living dimension | □ | □ | □ | □ | □ | □ | □ | □ | □ | □ | □ | □ | □ | □ | □ | □ | □ | Health dimension |

Q2: Please tick the relatively importance of each EAP factors in the “Creation Stage”.

| Stage | Intensity  Dimension | Absolutely important |  | Extremely  important |  | Quite important |  | Slightly important |  | Equally important |  | Slightly important |  | Quite important |  | Extremely  important |  | Absolutely important | Dimension |
| --- | --- | --- | --- | --- | --- | --- | --- | --- | --- | --- | --- | --- | --- | --- | --- | --- | --- | --- | --- |
|  |  | 9 | 8 | 7 | 6 | 5 | 4 | 3 | 2 | 1 | 2 | 3 | 4 | 5 | 6 | 7 | 8 | 9 |  |
| Creation | Work design | □ | □ | □ | □ | □ | □ | □ | □ | □ | □ | □ | □ | □ | □ | □ | □ | □ | Position change |
|  |  | □ | □ | □ | □ | □ | □ | □ | □ | □ | □ | □ | □ | □ | □ | □ | □ | □ | Retirement planning |
|  | Position change | □ | □ | □ | □ | □ | □ | □ | □ | □ | □ | □ | □ | □ | □ | □ | □ | □ |  |
|  | Family and marriage | □ | □ | □ | □ | □ | □ | □ | □ | □ | □ | □ | □ | □ | □ | □ | □ | □ | Interpersonal relationships |
|  |  | □ | □ | □ | □ | □ | □ | □ | □ | □ | □ | □ | □ | □ | □ | □ | □ | □ | Insurance planning |
|  |  | □ | □ | □ | □ | □ | □ | □ | □ | □ | □ | □ | □ | □ | □ | □ | □ | □ | Life assistance |
|  | Interpersonal relationships | □ | □ | □ | □ | □ | □ | □ | □ | □ | □ | □ | □ | □ | □ | □ | □ | □ | Insurance planning |
|  |  | □ | □ | □ | □ | □ | □ | □ | □ | □ | □ | □ | □ | □ | □ | □ | □ | □ | Life assistance |
|  | Insurance planning | □ | □ | □ | □ | □ | □ | □ | □ | □ | □ | □ | □ | □ | □ | □ | □ | □ |  |
|  | Worries and anxiety | □ | □ | □ | □ | □ | □ | □ | □ | □ | □ | □ | □ | □ | □ | □ | □ | □ | Healthy diet |
|  |  | □ | □ | □ | □ | □ | □ | □ | □ | □ | □ | □ | □ | □ | □ | □ | □ | □ | Mental health |
|  | Healthy diet | □ | □ | □ | □ | □ | □ | □ | □ | □ | □ | □ | □ | □ | □ | □ | □ | □ |  |
